# Changes in the levels of WBC count, PCT, CRP and ESR in Patients with acute Community-acquired Lower Respiratory tract infections and their diagnostic value

**DOI:** 10.12669/pjms.40.3.7699

**Published:** 2024

**Authors:** Na Li, Yinan Jia, Jianen Feng, Hu Chang, Siqi Li

**Affiliations:** 1 Na Li, Department of Infectious Diseases, Baoding NO.1 Central Hospital, Baoding 071000, Hebei, China; 2 Yinan Jia, Department of Internal Medical-Cardiovascular, Baoding NO.1 Central Hospital, Baoding 071000, Hebei, China; 3 Jianen Feng, The First Department of liver Diseases, Baoding People’s Hospital, Baoding 071000, Hebei, China; 4 Hu Chang, Department of Ophthalmology, Baoding NO.1 Central Hospital, Baoding 071000, Hebei, China; 5 Siqi Li, Department of Ophthalmology, Baoding NO.1 Central Hospital, Baoding 071000, Hebei, China

**Keywords:** Acute community-acquired lower respiratory tract infections, White blood cell count, Procalcitonin, C-reactive protein, Complications

## Abstract

**Objective:**

To investigate the levels of white blood cell count (WBC), procalcitonin (PCT), C-reactive protein (CRP) and erythrocyte sedimentation rate (ESR) in patients with acute community-acquired lower respiratory tract infections and the value of their combined detection in predicting the occurrence of complications.

**Methods:**

A retrospective analysis was conducted on the clinical data of 218 patients with acute community-acquired lower respiratory tract infections admitted to Baoding No.1 Central Hospital from January 2021 to December 2021. All patients were divided into two groups according to the presence of complications during treatment: the group with complications (observation group) and the group without complications (control group). The treatment situation of the two groups was compared, and their levels of WBC, PCT, CRP and ESR were quantitatively detected and compared.

**Results:**

Patients in the observation group were hospitalized for significantly longer days than those in the control group (*P*<0.05), and their combined pleural effusion percentage and oxygen uptake rate were higher than those in the control group (*P*<0.05). The levels of WBC, PCT, CRP and ESR in the observation group were significantly higher than those in the control group at admission, with statistically significant differences (*P*<0.05). Moreover, the positive rates of WBC, PCT, CRP and ESR in the observation group were higher than those in the control group in the single detection and the combined detection (*P*<0.05).

**Conclusions:**

The combined detection of WBC, PCT, CRP and ESR has substantial predictive value in predicting the occurrence of complications in patients with community-acquired lower respiratory tract infections.

## INTRODUCTION

Clinically, lower respiratory tract infections mainly refer to infections occurring in the respiratory tract below the glottis, including a large group of respiratory diseases such as pneumonia, bronchial asthma and bronchiectasis co-infection, acute bronchitis, chronic bronchitis co-infection, and acute exacerbation of the chronic obstructive pulmonary disease. They have pathogenic bacteria including chlamydia, mycoplasma, bacteria and viruses, etc., and are clinically manifested mainly by cough, expectoration and chest pain.^1^-^3^ Community-acquired lower respiratory tract infections, with recurrent clinical episodes, are quite common in lower respiratory tract infections. They may damage the immune system of patients and seriously threaten their quality of life and health. Therefore, community-acquired lower respiratory tract infections have become a significant problem of public health in China.^4^ With early diagnosis and aggressive treatment, patients with acute community-acquired lower respiratory tract infections can have improved treatment outcomes and a better prognosis.

In current clinical practice, the severity of acute community-acquired lower respiratory tract infections is mainly assessed based on the clinical manifestations and etiological examination of patients. The specificity of clinical symptom criteria is low; Etiological examination, although highly specific, requires relevant laboratory cultures and takes a long time with delayed results, which may delay the diagnosis.^5^ Acute community-acquired lower respiratory tract infections give rise to an inflammatory response, while white blood cell (WBC), procalcitonin (PCT), C-reactive protein (CRP) and erythrocyte sedimentation rate (ESR) are crucial indicators of the inflammatory response.^6^ The present study aimed to investigate the changes in the levels of WBC, PCT, CRP and ESR in patients with acute community-acquired lower respiratory tract infections and their diagnostic value for the severity of the disease.

## METHODS

A retrospective analysis was conducted on the clinical data of 218 patients with acute community-acquired lower respiratory tract infections admitted to Baoding No.1 Central Hospital from January 2021 to December 2021. All patients were divided into two groups according to the presence of complications during treatment: the group with complications (observation group) and the group without complications (control group). In the observation group, there were 118 cases including 79 males and 39 females, aged 18-99 years, with a mean of (63.38±17.51) years. In the control group, there were 100 cases including 54 males and 46 females, aged 18-89 years, with an average of (59.28±17.49) years. No statistically significant differences were observed between the two groups in terms of age and gender (*P*>0.05).

### Ethical Approval:

The study was approved by the Institutional Ethics Committee of Baoding No.1 Central Hospital (No.: [2022]047; November 3, 2022), and written informed consent was obtained from all participants.

### Inclusion criteria:

- Patients meeting the diagnostic criteria of acute community-acquired lower respiratory tract infections;
- Patients with complete clinical data.

### Exclusion criteria:

- Patients with expression disorders or concomitant psychiatric disorders;
- Patients with congenital heart disease;
- Patients with other infectious diseases, such as cardiopulmonary resuscitation, trauma, post-operation, burn, shock, sunstroke, neuroendocrine neoplasm, extracorporeal circulation, liver cirrhosis, pancreatitis, mesenteric necrosis and catheter infections;
- Patients with incomplete clinical data.

All patients were given anti-infective treatment based on their condition and drug sensitivity after being diagnosed in our hospital. Peripheral venous blood 5ml was collected from patients on admission to detect their WBC, PCT, CRP and ESR. Specifically, WBC was detected by a five-classification fully automatic hematology analyzer with a reference range of (4-10) ×10^9^/L, and a value higher than 10×10^9^/L was considered abnormal. PCT was detected by an automatic biochemical analyzer with a reference range of <0.5 μg/L, and a value higher than 0.5μg/L was considered abnormal. CRP was detected by immunoturbidimetric method with a reference range of 0-10 mg/L, and a value higher than 10 mg/L was considered abnormal. ESR was detected by the Westergren method with a reference range of <15 mm/h for males and <20 mm/h for females, and values higher than these were considered abnormal. All patients in this study were routinely examined for blood analysis, urine analysis and chest radiographs. For patients with abnormal body temperature, examinations such as erythrocyte sedimentation rate and chest CT were added.

### Observation indexes:

The levels of WBC, PCT, CRP and ESR at admission were compared between the two groups.

All patients were divided into the mild group and the severe group according to the severity of the disease, and the levels of WBC, PCT, CRP and ESR in patients with different severity of the disease were compared.

Pearson correlation analysis was employed to analyze the correlation of serum WBC, PCT, CRP and ESR at admission in patients with acute community-acquired lower respiratory tract infections.

The receiver operating curve (ROC) was plotted to evaluate the predictive value of WBC, PCT, CRP and ESR levels in patients with acute community-acquired lower respiratory tract infections.

### Statistical Analysis:

All data in this study were statistically analyzed using SPSS22.0 software. Measurement data were expressed as (*x̅*±*S*), and an independent sample *t* test was used for comparison between the two groups before and after treatment. Enumeration data were expressed as n (%), and the c² test was used for comparison between the two groups. The power of test / confidence interval is 95%. Logistic regression equations were used for multivariate analysis of the severity of the disease, with P<0.05 indicating a statistically significant difference.

## RESULTS

The length of hospitalization in the observation group was significantly longer than that in the control group, with a statistically significant difference (*P*<0.05). The percentage of combined pleural effusion in the observation group was 38.14%, which was higher than that in the control group (21.00%), with a statistically significant difference (*P*<0.05). Moreover, 82.20% of patients in the observation group required oxygen uptake during treatment, which was higher than 38.00% in the control group, with a statistically significant difference (*P*<0.05), Table-I.

**Table-I:** Comparative analysis of condition-related conditions in the two groups.

Group	n	Interval from onset of symptoms to hospitalization (days) (χ̅±S)	Fever (Yes/No, n)	Dyspnea (Yes/No, n)	Smoking (Yes/No, n)	Length of stay (days) (χ̅±S)	Pleural effusion (Yes/No, n)	Oxygen uptake or not (Yes/No, n)
Observation group	118	4.49±2.23	89/29	60/58	45/73	11.85±5.62	45/73	97/21
Control group	100	4.38±2.09	65/35	61/39	33/67	9.12±3.48	21/79	38/62
*t/χ^2^* value		0.378	2.836	2.259	0.621	4.213	7.529	44.858
*P* value		0.706	0.092	0.133	0.431	0.000	0.006	0.000

The levels of WBC, PCT, CRP and ESR in the observation group at admission were significantly higher than those in the control group, with statistically significant differences (*P*<0.05), Table-II. The positive rates of WBC, PCT, CRP and ESR in the observation group were higher than those in the control group in the single detection and the combined detection (*P*<0.05), Table-III.

**Table-II:** Comparative analysis of the levels of WBC, PCT, CRP and ESR in the two groups (*χ̅*±*S*).

Group	n	WBC (×10^9^/L)	PCT (μg/L)	CRP (g/L)	ESR (mm/h)
Observation group	118	10.54±5.53	3.09±12.30	104.66±87.48	59.18±39.64
Control group	100	8.34±4.26	0.18±0.53	51.81±56.55	36.06±28.72
*T* value		3.104	2.364	5.191	4.851
*P* value		0.002	0.019	0.000	0.000

**Table-III:** Comparative analysis of the positive rates of WBC, PCT, CRP and ESR in the two groups (n, %).

Group	n	Positive rate of WBC	Positive rate of PCT	Positive rate of CRP	Positive rate of ESR	Positive rate of combined detection
Observation group	118	57 (48.31)	42 (35.59)	100 (84.75)	110 (93.22)	112 (94.92)
Control group	100	25 (25.00)	14 (14.00)	61 (61.00)	73 (73.00)	81 (81.00)
c^2^ value		12.528	13.221	15.806	16.421	10.323
*P* value		0.000	0.000	0.000	0.000	0.001

A multivariate Logistic regression analysis was performed with the presence of complications as the dependent variable and WBC, PCT, CRP and ESR as the independent variables. The results showed that elevated CRP and ESR were independent risk factors for complications in acute community-acquired lower respiratory tract infections (*P*<0.05). Table-IV.

**Table-IV:** Multivariate Logistic regression analysis of factors influencing the occurrence of complications of acute community-acquired lower respiratory tract infections.

Factor	Regression coefficient	Standard error	Wald value	P	OR	95%CI
WBC	0.540	0.331	2.662	0.103	0.583	0.305-1.115
PCT	0.540	0.373	2.094	0.148	0.583	0.281-1.211
CRP	1.108	0.470	5.551	0.018	0.330	0.131-0.830
ESR	0.012	0.005	6.997	0.008	0.989	0.979-0.997

**Fig.1:**
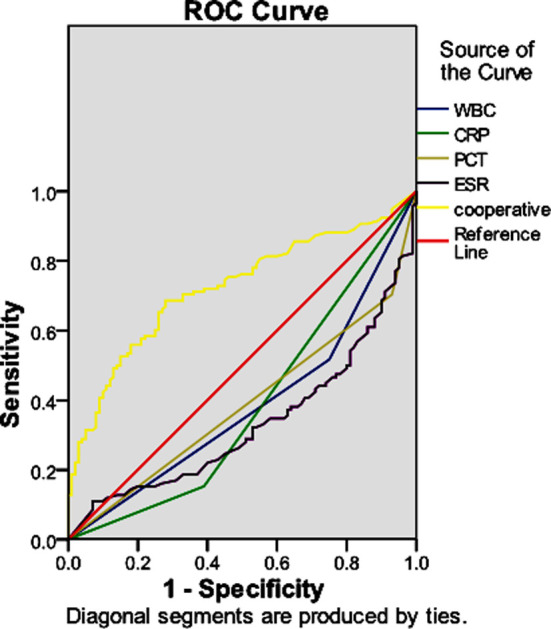
ROC curve of the predictive value of WBC, PCT, CRP and ESR.

Predictive value of single detection and combined detection of WBC, PCT, CRP and ESR levels in predicting the occurrence of complications in acute community-acquired lower respiratory tract infections. ROC curve analysis showed that the area under the ROC curve of WBC, PCT, CRP and ESR in the combined detection was significantly higher than that in the single detection, Fig.1.

## DISCUSSION

In this study, the levels of WBC, PCT, CRP and ESR showed the same trend of change as the severity of patients’ disease, indicating that the levels of WBC, PCT, CRP and ESR in patients with complications were higher than those in those without complications. Multivariate *Logistic* regression analysis showed that elevated PCT, CRP and ESR were independent risk factors for complications of acute community-acquired lower respiratory tract infections (*P*<0.05). ROC curve showed that the area under the curve, sensitivity and specificity of combined detection of all indicators were significantly higher than that of single detection, suggesting that the levels of WBC, PCT, CRP and ESR could be used in the diagnosis of acute community-acquired lower respiratory tract infections. The sensitivity and specificity of the diagnosis are high when applying the combined detection of all indicators, and it is beneficial to determine the severity of the disease.

PCT is a peptide precursor of calcitonin, which consists of 116 amino acids. It is transcribed and produced in parafollicular cells of the thyroid gland and is stable and easily detectable. Physiologically, PCT is not released into the bloodstream. Therefore, the serum level of PCT in healthy people is extremely low, mostly <0.5 μg/L, while more than 0.5 μg/L can be considered a significant infection in the organism. The level of PCT has a close bearing on the severity of infection. It was reported in the literature^7^ that the level of PCT can be used to assess and monitor the disease condition and also demonstrate high clinical value in determining the prognosis of the disease. In case of infection in the organism, PCT can be elevated after two to three hours of stimulation by inflammatory factors, peaking at about 24 hours of infection. PCT is specifically elevated in patients with bacterial infections, while it is generally not elevated in non-bacterial infections. Therefore, PCT has a high specificity and sensitivity for the diagnosis of bacterial infections and can be detected early. In this study, the level of PCT in the observation group was significantly higher than that in the control group, indicating that PCT can respond quickly to the inflammatory response of the body, especially the severe inflammatory response, with a higher level of increase.

Acute community-acquired lower respiratory tract infections are currently common respiratory infectious diseases, with an increasing incidence year by year. Clinically, this disease is mainly diagnosed by clinical symptoms, signs, chest radiographs and lung CT.^8^,^9^ However, the disease is difficult to differentially diagnose because of the absence of typical clinical symptoms and signs.^10^ To this end, timely and accurate diagnosis of acute community-acquired lower respiratory tract infections is crucial to the prognosis after clinical treatment.^11^ The changes in white blood cell count (WBC) and the expression of inflammatory markers are obviously correlated with organism infection, which are important indicators for determining the inflammatory response of the organism. They are of great clinical value in determining the presence and severity of inflammatory response in the organism. Clinically, the most widely used indicators are WBC, PCT, CRP and ESR.^12^-^14^

WBC in blood routine is susceptible to physiological factors and environmental factors, such as smoking, physical labor and strenuous exercise, and will increase reflexively but to a limited extent.^15^ At present, WBC is commonly used in the observation of various infections. In case of bacterial infections in the organism, the WBC level will increase rapidly with a positive correlation to the degree of infection. The WBC level is also elevated in some non-bacterial infections while normal or lower in viral or other infections. For this reason, WBC alone has high specificity but low sensitivity in the diagnosis of inflammatory reactions.^16^ In this study, complications occurred in the observation group during clinical treatment, indicating a severe inflammatory response, and the WBC level was significantly higher than that of the control group, consistent with those reported in the clinical literature.

Currently, an important indicator for the diagnosis of acute community-acquired lower respiratory tract infections is the detection of CRP levels. CRP, an acute phase reaction protein (APRP), has very low levels in the physiological state and its levels can rise rapidly during the inflammatory response, peaking at about 36-50 hours. Therefore, CRP is another indicator for early diagnosis of infection besides PCT. However, it has some limitations compared to PCT, such as its ability to be elevated even in non-infected and stressed states, and it is only clinically meaningful to be elevated 12 hours after the onset of inflammation, which affects its clinical specificity.^17^ A study showed^18^ a positive correlation between CRP levels and the degree of infection. In the present study, CRP levels in the observation group with complications were significantly higher than those in the control group without complications, which is consistent with clinical studies.

ESR is a non-specific indicator for the diagnosis of inflammatory diseases and can lead to overlapping red blood cells in the presence of inflammation in the organism. As a result, the area of resistance of red blood cells is reduced, leading to an increase in ESR levels. However, changes in ESR levels are influenced by various factors, such as the number of red blood cells, hemoglobin content, triglyceride levels, etc. It is usually not used for clinical diagnosis alone but is often applied in combination with other markers such as routine blood and CRP levels. In this study, the ESR levels in the observation group with complications were significantly higher than those in the control group without complications.

### Limitations of this study:

It includes small sample size and the lack of follow-up are two major limitations of our study. In addition, we only analyzed and discussed the cases included in our hospital, which may not be representative enough. We look forward to a multi-center study in the future to reach more comprehensive conclusions.

## CONCLUSIONS

Patients with acute community-acquired lower respiratory tract infections combined with complications tend to have pleural effusions during treatment, require oxygen uptake, and have long hospital stays. These patients have mostly significantly higher levels of WBC, PCT, CRP and ESR than those without complications. The combined detection of WBC, PCT, CRP and ESR has substantial predictive value in predicting the occurrence of complications in patients with community-acquired lower respiratory tract infections, which is worth promoting in clinical practice.

### Authors’ Contributions:

**NL** and **YJ** carried out the studies, participated in collecting data, drafted the manuscript, are responsible and accountable for the accuracy and integrity of the work. **JF** performed the statistical analysis and participated in its design. **HC** and **SL** participated in acquisition, analysis, interpretation of data and draft of the manuscript. All authors read and approved the final manuscript.
